# Proteomic Analysis of Human Serum from Patients with Chronic Kidney Disease

**DOI:** 10.3390/biom10020257

**Published:** 2020-02-07

**Authors:** Yulia Romanova, Alexander Laikov, Maria Markelova, Rania Khadiullina, Alfiz Makseev, Milausha Hasanova, Albert Rizvanov, Svetlana Khaiboullina, Ilnur Salafutdinov

**Affiliations:** 1 Institute of Fundamental Medicine and Biology, Kazan Federal University, 420008 Kazan, Tartastan, Russia; alexander.laikov@yandex.ru (A.L.); mimarkelova@gmail.com (M.M.); nazyrova.95@yandex.ru (R.K.); rizvanov@gmail.com (A.R.); 2 Republican Clinical Hospital Ministry of Health Republic of Tatarstan, 420064 Kazan, Tatarstan, Russia; alfiz.mar82@inbox.ru (A.M.); milyash@inbox.ru (M.H.); 3 Department of Urology and Nephrology, Kazan State Medical Academy, 420012 Kazan, Tatarstan, Russia; 4 Department of Microbiology and Immunology, University of Nevada, Reno, NV 89557, USA; sv.khaiboullina@gmail.com

**Keywords:** chronic kidney disease, inflammation, cytokines, biomarkers, 2D-DIGE, multiple reaction monitoring

## Abstract

Chronic kidney disease (CKD) is an important public health problem in the world. The aim of our research was to identify novel potential serum biomarkers of renal injury. ELISA assay showed that cytokines and chemokines IL-1β, IL-2, IL-4, IL-5, IL-6, IL-7, IL-8, IL-9, IL-10, IL-12 (p70), IL-13, IL-15, IL-17, Eotaxin, FGFb, G-CSF, GM-CSF, IP-10, MCP-1, MIP-1α, MIP-1β, PDGF-1bb, RANTES, TNF-α and VEGF were significantly higher (R > 0.6, *p* value < 0.05) in the serum of patients with CKD compared to healthy subjects, and they were positively correlated with well-established markers (urea and creatinine). The multiple reaction monitoring (MRM) quantification method revealed that levels of HSP90B2, AAT, IGSF22, CUL5, PKCE, APOA4, APOE, APOA1, CCDC171, CCDC43, VIL1, Antigen KI-67, NKRF, APPBP2, CAPRI and most complement system proteins were increased in serum of CKD patients compared to the healthy group. Among complement system proteins, the C8G subunit was significantly decreased three-fold in patients with CKD. However, only AAT and HSP90B2 were positively correlated with well-established markers and, therefore, could be proposed as potential biomarkers for CKD.

## 1. Introduction

Chronic kidney disease (CKD) is defined by a gradual loss of kidney function for more than three months. CKD remains an increasing public health threat affecting up to 16% of the global population [1]. According to the 2016 Global Burden of Disease study, CKD caused 16.9 deaths per 100,000 in 2016 [2]. CKD is characterized by progressive kidney damage due to renal circulatory impairment, which is common for patients diagnosed with diabetes and hypertension. The treatment is not specific and aimed to improve the underlying condition as well as to manage complications.

The initial symptoms of CKD are not specific, which prevents early diagnosis. Diagnosis of CKD is based on the calculation of glomerular filtration rate (GFR), detection of albuminuria and a kidney biopsy [3,4]. These diagnostic methods are often used at the late stage of disease, when kidney damage is advanced and often irreversible. Therefore, identification of biomarkers for early diagnosis of CKD is important. Several markers of kidney inflammation were proposed for CKD diagnosis including plasma and urine interleukins and chemokines (IL-18, IL-10, IL-6, MCP-1, FGF-23) [5,6]. Also, to improve predicting CKD prognosis, the biomarkers of renal tubular injury NGAL, KIM-1, TIMP-2 and IGFBP-7 have been shown to be especially promising [7,8]. Development of novel genetic tests and identification of genomic biomarkers can also improve the management of CKD by facilitating early diagnosis and advancing patient management [9,10]. Nevertheless, investigation of novel and effective biomarkers for early CKD diagnosis remains an important aim.

Changes in blood proteins are commonly monitored to diagnose the disease, mainly because of their easy access. Also, changes in blood components can be detected in the early stage of the disease. This is especially true in CKD, where changes in kidney filtration result in significant alterations in blood chemistry. For example, increased serum creatinine and urea levels, electrolyte imbalance and increased blood coagulation are found in CKD patients. Therefore, we suggest that potential CKD biomarkers might be identified in patient serum.

Previous studies of novel, early-stage CKD biomarkers have used different proteomic approaches [11,12,13,14]. We applied a two-dimensional fluorescence difference gel electrophoresis (2D-DIGE) approach to separate depleted serum proteins in CKD and control serum. High-abundance proteins were deleted to improve visualization of protein spots. After differentially expressed proteins were identified, these proteins were quantified with multiple reaction monitoring (MRM) in the serum samples of patients (n = 26) and control groups (n = 10).

## 2. Materials and Methods

### 2.1. Subjects

Serum samples from 26 adult patients with terminal stage CKD (18 male and 8 female; age 53.0 ± 16.2 y) and 10 control healthy subjects older than 18 y (4 male and 6 female; age 29 ± 6.7 y) were used in this study. CKD patients not treated with hemodialysis were recruited from the Department of Nephrology, Republican Clinical Hospital Ministry of Health Republic of Tatarstan. The diagnosis of CKD was confirmed by clinical tests (eGFR < 15 mL/min/1.73 m^2^, protein in urea > 0.033 g/L, serum creatinine > 115 mmol/L, etc.). Baseline clinical characteristics and laboratory data of the study population are summarized in Table 1. Serum samples were stored at −80 °C until they were used.

### 2.2. Ethics Statement

The Ethics Committee of the Kazan Federal University approved this study (protocol N4/09), and informed written consent was obtained from each patient and control according to the Guidelines approved under this Protocol (article 20, Federal Law “Protection of Health Right of Citizens of Russian Federation” N323- FZ, 11.21.2011).

### 2.3. 2D-DIGE of Serum Proteins after Depletion

Unspecific depletion of high-abundance proteins in serum samples (200 μL) was done using a commercially available kit, ProteoMiner (Bio-Rad, Hercules, CA, USA), according to the manufacturer’s instructions with a minor modification. Briefly, 200 μL of serum was loaded into the prepared spin column with protein-binding sorbent, incubated for 2 h at room temperature, and then washed with PBS (150 mM NaCl, 10 mM NaH2PO4, pH 7.4) and water. Finally, the protein was eluted using a denaturing buffer (8 М urea, 10 mМ Tris-HCl, рН 8.5, 3% CHAPS (3-[(3-Cholamidopropyl)dimethylammonio]-1-propanesulfonate hydrate), 2% NP40 (nonylphenol ethoxylate)) and used for 2D-DIGE.

Serum proteins were separated using 2D-DIGE as previously described [15]. Isoelectric focusing (IEF) was done with commercially available 17 cm IPG (immobilized pH gradient) strips in the pH range 3–10 (Bio-Rad, Hercules, CA, USA) according to the manufacturer’s instructions. We used 150 μg of each protein sample to label with 0.4 mM cyanine dyes Cy3 or Cy5 (Lumiprobe, Moscow, Russia) before IEF. The samples were mixed, diluted in the rehydration buffer (8 М urea, 10 mМ Tris-HCl (рН 8.5), 3% CHAPS, 2% NP40, 50 mM DTT (dithiothreitol), 0.4% Pharmalyte, pH range 3–10 (GE Healthcare, Chicago, IL, USA)) and loaded into the strip for active rehydration for 12 h. Isoelectric focusing was carried out using the Protean i12 IEF cell (Bio-Rad, Hercules, CA, USA). After IEF, the strips were incubated for 10 min in the equilibration buffer (6 M urea, 0.375 M Tris-HCl, pH 8.8, 2% SDS (sodium dodecyl sulfate)), containing 2% DTT, and for 10 min in the same equilibration buffer, containing 2.5% iodoacetamide. Second-dimension electrophoresis was performed on self-cast gradient 9%–16% polyacrylamide gels. Gels were scanned with 100 μm pixel resolution using the Typhoon FLA 9500 scanner (GE Healthcare, Chicago, IL, USA). Then, the gels were stained with silver nitrate [16].

### 2.4. Protein Identification 

Collected protein spots were destained using ferricyanide-thiosulfate as previously described [17] with some modifications. Briefly, gel pieces were washed with Milli-Q water and destained using a 30 mM potassium ferricyanide and 100 mM sodium thiosulfate solution for 5 min. Then, the gel pieces were rinsed three times with Milli-Q water, dehydrated using acetonitrile, and digested overnight (0.1% trypsin (MS grad, Promega, Madison, WI, USA) at 37 °C). Peptides were extracted using 0.2% trifluoroacetic acid (TFA) followed by incubation in 100% acetonitrile (ACN) for 30 min. Collected peptides were dried at 45 °C for 2 h, dissolved in 5 μL of 0.2% TFA and analyzed by a MALDI-TOF mass spectrometer Ultraflex (Bruker Daltonics GmbH, Bremen, Germany). The spectra were recorded in positive reflector mode from 700 to 3500 *m*/*z*. Peptide mass fingerprinting (PMF) was performed using the MASCOT software for searching matches in Swiss-Prot and NCBI databases.

### 2.5. Preparation of Serum Tryptic Digests

Tryptic digestion was carried out manually as previously described [18] with some modifications. Serum (8 μL) was diluted in 196.5 μL of 25 mM ammonium bicarbonate before protein denature by adding 30 μL of 10% sodium deoxycholate. Then, samples were reduced (26.1 μL of 50 mM tris(2-carboxyethyl)phosphine (TCEP) at 60 °C for 30 min) and alkylated (29.0 μL of 100 mM iodoacetamide; at 37 °C for 30 min in the dark). The remaining iodoacetamide was quenched by adding 29.0 μL of 100 mM DTT (37 °C; 30 min). Each sample was digested with 4 μL of 0.1% trypsin (MS grad, Promega, Madison, WI, USA) at 37 °C for 16 h. Digestion was quenched using formic acid at a final concentration 0.5% *v*/*v*. Samples were centrifuged (10 min at 10000× *g*), and supernatants were desalted and concentrated using solid phase extraction (Discovery DSC-18 (50 mg) cartridges (Supelco, Bellefonte, PA, USA)) according to the manufacturer’s protocol. Eluted peptides were dried at 45 °C for several hours and rehydrated (40 μL of 0.1% *v*/*v* formic acid in water/acetonitrile (95/5)) prior to LC-MRM/MS analysis.

### 2.6. LC-MRM/MS Analysis of Serum Digests

Peptides (10 μL) were separated in a reversed phase analytical column (100 × 2.1 mm i.d., Titan C18, 1.9 μm particle size (Supelco, Bellefonte, PA, USA)) with an Agilent 1290 Infinity UHPLC system coupled to a QTRAP 6500 (AB Sciex, Darmstadt, Germany) mass spectrometer. Proteins were separated using 400 μL/min flow rate and a gradient from 5%–95% mobile phase B, temperature 40 °C and a 25 min total run time. Mobile phase A consisted of 95% 0.1% *v*/*v* formic acid in water and 5% ACN, and mobile phase B consisted of 95% ACN and 5% 0.1% formic acid in water. The linear gradients were as follows (time: % B): 0.3 min: 5% B; 17 min: 40% B; 18 min: 95% B; 21.5 min: 95% B; 23 min: 5% B; 25 min: 95% B. All acquisition methods used the following parameters: 5200 V capillary voltage; source type Turbo Spray Ion Drive with temperature 500 °C; curtain gas 35 psi; declustering potential 51 V; collision energy was automatically optimized for each transition; flow rate 0.4 mL/min. Mass spectrometric data were analyzed using MultiQuant 3.0.2 software (AB Sciex, Darmstadt, Germany).

Skyline 3.6.0 [19] was used to generate precursor/fragment ion pairs, so-called MRM transitions, in silico [20]. The following options were selected: the peptide length was set to 8–25 amino acids, no post-translational modification (PTM) and one missed cleavage was allowed. In addition, two or three charge states of peptides were chosen for further MRM experiments. At least two peptides were chosen for identification of the target protein (Supplementary Table S1). The MRM method included at least three MRM transitions per peptide to select the best transition. Data analysis was done, and the areas for all the transitions were calculated using the Analyst 1.6.2 and MultiQuant 3.0.2 software (AB Sciex, Darmstadt, Germany). Used peptides with unique sequences and scheduled MRM transitions are given in Table 2.

### 2.7. Analysis of Serum Levels of Cytokines

Quantitative analyses of cytokines (IL-1β, IL-2Rα, IL-2, IL-4, IL-5, IL-6, IL-7, IL-8, IL-9, IL-10, IL-12 (p70), IL-13, IL-15, IL-17), Eotaxin, basic fibroblast growth factor (FGFb), granulocyte colony-stimulating factor (G-CSF), granulocyte macrophage colony-stimulating factor (GM-CSF), interferon gamma (INFγ), interferon gamma-induced protein 10 (IP-10), mast cell proteinase-1 (MCP-1), macrophage inflammatory protein 1-alpha (MIP-1α), macrophage inflammatory protein 1-beta (MIP-1 β), platelet-derived growth factor with two B subunits (PDGF-1bb), chemokine RANTES, tumor necrosis factor beta (TNF-β) and vascular endothelial growth factor (VEGF) in blood serum were performed using the multiplex analyzer Bio-Plex200 System (BioRad, Hercules, CA, USA) and “Bio-Plex Pro™ Human Cytokine 27-plex Assay” kit (BioRad, Hercules, CA, USA) according to the manufacturer‘s recommendations.

### 2.8. Statistical Analysis

Statistical analysis of the multiple reaction monitoring (MRM) data and ELISA data were performed in the R environment [21]. Statistically significant differences between groups of patients and healthy individuals were accepted as *p* < 0.05, assessed by the Wilcoxon rank sum test with Benjamini–Hochberg adjustment for MRM data and *p* < 0.01 for ELISA data. Correlations between the concentrations of serum proteins, cytokines, urea and creatinine were analyzed using the R Hmisc package (based on Spearman’s rank correlation coefficient).

## 3. Results

**Figure 1:**
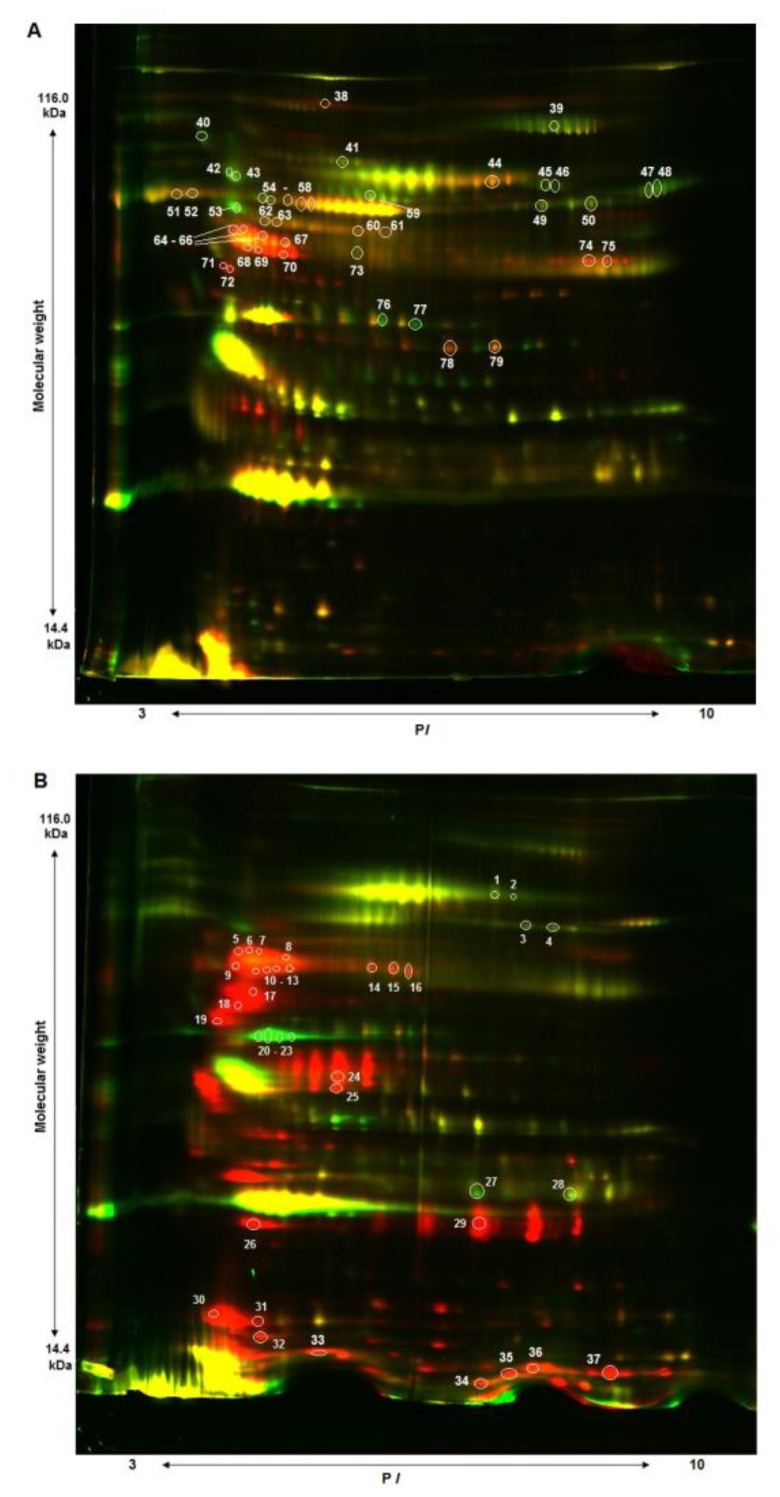
2D-DIGE protein profiles of depleted serum of patients with chronic kidney disease (CKD; red fluorescent dye) and healthy individuals (green fluorescent dye). (**A**) serum of two women, 30 and 35 y. (**B**) serum of two men, 34 and 32 y.

Two-dimensional fluorescence difference gel electrophoresis (2D-DIGE) is a convenient method to identify differences in protein profiles of the samples compared; however, it imposes a number of requirements on an analyte. In particular, successful separation and visualization of protein spots in gel depend upon concentrations of the proteins analyzed. The serum proteome has a dynamic range of more than ten orders of magnitude; thus, there is an excess of major proteins of albumin (more than 60% of the total amount of proteins), alpha-, beta- and gamma-globulin fractions (over 30%), with the rest being less than 10% of the total number of serum proteins [4,22,23]. Prior to 2D gel-electrophoresis, samples were depleted with the use of a commercial “ProteoMiner” kit (Bio-Rad) in order to remove major proteins. As can be seen in Figure 1, removal of most of the major fraction facilitated better blood serum protein separation. Protein spots with different expression levels between two patients and two healthy subjects were cut off and identified; these data are presented in Supplementary Table S2. Some proteins were presented by several spots on 2D gel, probably being isoforms of the same protein.

Fifty-six unique, differentially expressed proteins between CKD patients and healthy subjects were identified with MALDI mass spectrometry, and we surmised there might be potential diagnostic markers of early CKD stages among these proteins. To test this hypothesis, 20 proteins with differential expression were quantified in the serum of CKD patients (n = 26) and healthy volunteers (n = 10) using MRM (multiple reaction monitoring). At present, MRM is an advanced method of mass spectrometry and allows simultaneous quantitation of numerous protein concentrations by the signal intensity of daughter ions, being fragments of known parent peptides [24]. For this purpose, we analyzed amino acid sequences of chosen proteins and selected relevant peptides to quantify proteins of interest using Skyline software (Table 2).

Two apolipoproteins, APOA1 and APOA4, were included in the list of proteins selected for MRM, with APOE added as a component that plays an important role in lipid metabolism and is associated with impaired hemodialysis and renal transplant functioning. Moreover, 18 complement system proteins were added into the expanded list, as they were directly involved in inflammatory reactions (Table 2).

**Figure 2:**
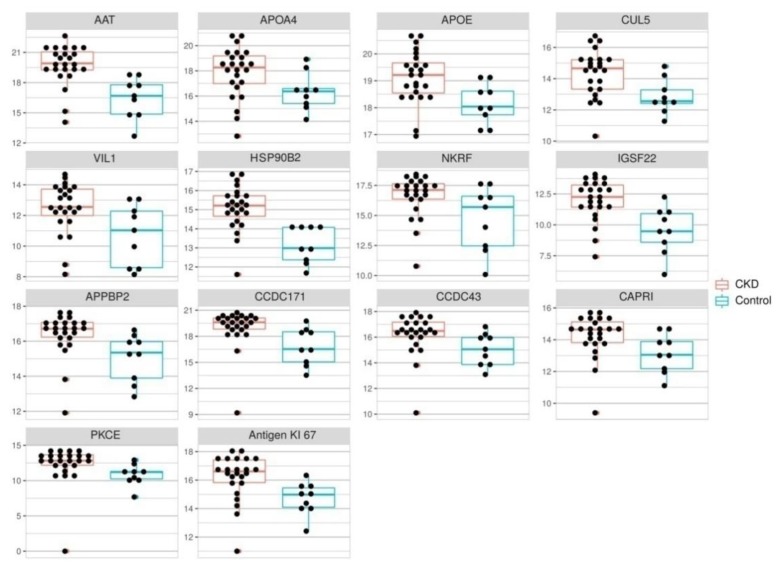
Dot plots of complement system components levels in the serum of healthy individuals (n = 10, yellow) and patients with CKD (n = 26, blue). Levels are expressed as areas of MRM transition peaks. Wilcoxon rank sum test was performed in each case, *p* value < 0.05.

**Figure 3:**
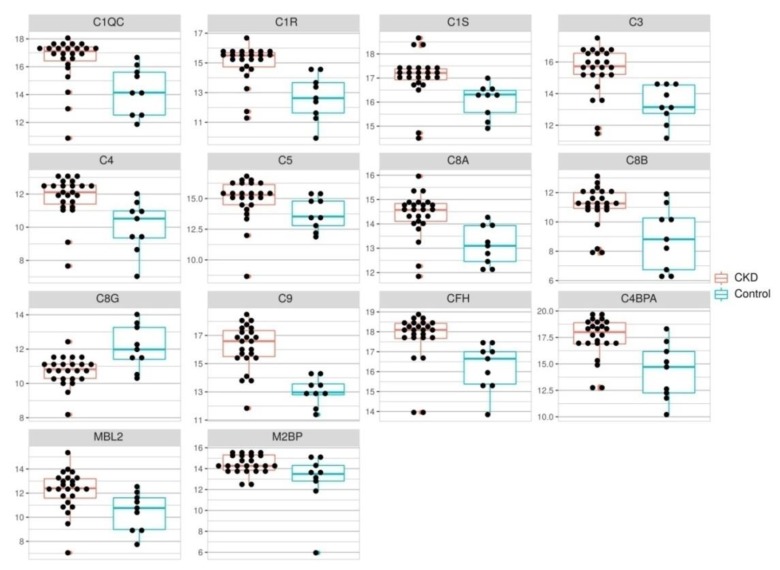
Dot plots of differently expressed protein levels in the serum of healthy individuals (n = 10, yellow) and patients with CKD (n = 26, blue). Levels are expressed as areas of MRM transition peaks. Wilcoxon rank sum test was performed in each case, *p* value < 0.05.

The quantification results for proteins with differential expression in 2D-DIGE and those associated with the complement system are shown in Figure 2 and Figure 3.

In general, concentrations of 26 proteins enlisted in Table 2 were significantly different in patients with CKD and healthy subjects (Figure 2 and Figure 3, Supplementary Tables S3 and S4). IGSF22, HSP90B2, AAT, C4BPA, C3, C1R and C9 concentrations increased more than four times in the group of CKD patients as compared to the control. Serum CFH, CUL5, PKCE, APOA4, APOE, CCDC171, CCDC43, VIL1, antigen KI-67, NKRF, APPBP2, CAPRI, C1QC, C1S, C4, C5, C8A, C8B and MBL2 concentrations were two to three times elevated in patients with CKD. Concentrations of the remaining proteins such as CCT4, PLG, APOA1, LMCD1, CKAP2L, PLB1, PPEF2, Gal-3, MASP2, C6 and FCN3 were not significantly different between the group of CKD patients and healthy subjects. Only the concentration of C8G was significantly decreased (three-fold) in patients with CKD.

**Figure 4:**
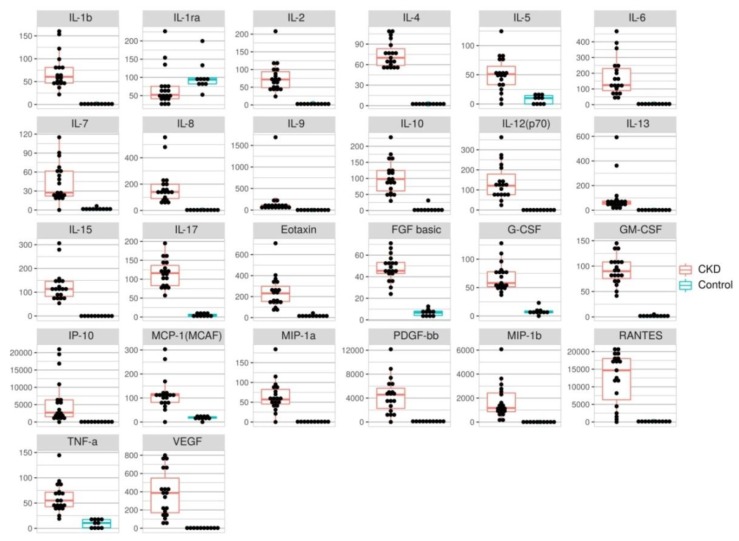
Dot plots of serum levels (pg/mL) of 26 cytokines for healthy people (n = 10, red) and patients with CKD (n = 19, blue). Wilcoxon rank sum test was performed in each case, *p* value < 0.01.

To assess the immune status serum concentrations of 27 cytokines, chemokines and growth factors such as IL-1β, IL-2Rα, IL-2, IL-4, IL-5, IL-6, IL-7, IL-8, IL-9, IL-10, IL-12 (p70), IL-13, IL-15, IL-17, Eotaxin, FGFb, G-CSF, GM-CSF, INFγ, IP-10, MCP-1, MIP-1α, MIP-1β, PDGF-1bb, RANTES, TNF-α and VEGF, they were quantified using Luminex xMAP technology (Figure 4). The analysis showed that serum concentrations of IL-9 and MIP-1β were elevated >500 times; those of IL-1β, IL-8, IL-12 (p70), IL-13, IL-15, IP-10, RANTES and VEGF increased >100 times; IL-6, GM-CSF, MIP-1α and PDGF-1bb levels were elevated >50 times; IL-2, IL-4, IL-7, IL-10 and IL-17 were 20–40 times higher; and those of IL-5, Eotaxin, FGFb, G-CSF, MCP-1 and TNF-α increased 6–12 times in patients with CKD (n = 19) when compared to the control group (n = 10). The INFγ concentration did not significantly differ between the groups. The blood serum concentration of soluble IL-2Rα was reduced 1.5 times in patients versus the control group.

**Figure 5:**
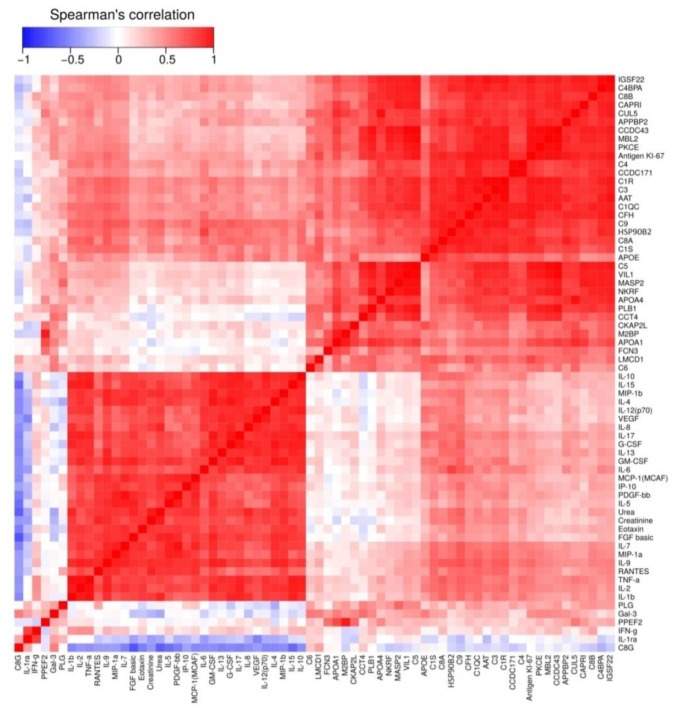
Heat map demonstrating correlations between urea, creatinine, cytokines and serum proteins. Hierarchical clustering of all analytes was performed by using the Euclidian distance method. The blue and red colors represent negative and positive Spearman’s rank correlation coefficients between the two analytes, respectively.

As concentrations of most proteins and cytokines analyzed were significantly different in the group of CKD patients and healthy subjects, clustering of quantitative serum protein proteomic analysis results, cytokine multiplex analysis data and creatinine and urea biochemistry results were compiled (Figure 5, Supplementary Table S5). As can be seen from the heat map data, an increase of all analyzed cytokines, except for IL-2Rα and INFγ, positively correlated with serum creatinine and urea concentrations (R > 0.6, *p* value < 0.05). Serum AAT concentration demonstrated a moderate, positive correlation (R = 0.44, *p* value < 0.05) with creatinine and some cytokines (IL-2, IL-6, IL-7, IL-9, MIP-1α, RANTES; R > 0.5, *p* value < 0.05). HSP90B2 positively correlated with those of creatinine and urea and most of the measured cytokines (IL-2, IL-6, IL-7, IL-8, IL-9, IL-10, IL-13, IL-17, G-CSF, GM-CSF, MIP-1α, MIP-1β, RANTES, TNF-α and VEGF; R > 0.5, *p* value < 0.05). Serum APOE, IGSF22, CUL5, CCDC171, CAPRI, CCDC43, Antigen KI-67, PKCE and APPBP2 levels positively correlated only with those of some cytokines such as IL-2, IL-9, MIP-1a, TNF-α and/or IL-13, IL-17, RANTES (R > 0.4, *p* value < 0.05). Serum APOA4, APOA1, VIL1 and NKRF concentrations had no correlations with creatinine, urea and cytokines.

Complement system components such as CFH, C1S, C1R, C1QC, C3, C4, С8A and C9 had a positive correlation with serum urea and/or creatinine levels (R > 0.4, *p* value < 0.05), as well as with cytokines (IL-2, IL-7, IL-9, IL-17, MIP-1α, RANTES and TNF-α; R > 0.45, *p* value < 0.05). MBL2 levels mildly correlated with some cytokines (IL-2, IL-9, MIP-1a, RANTES; R < −0.45, *p* value < 0.05). C8G had a negative correlation with creatinine (R = −0.63, *p* value < 0.05) and most cytokines (IL-2, IL-4, IL-5, IL-6, IL-7, IL-8, IL-10, IL12(p70), IL-13, IL-15, IL-17, G-CSF, Eotaxin, FGFb and MIP-1β; R < −0.45, *p* value < 0.05). The remaining complement system proteins (i.e., C5, C6, Gal3, MASP2 and M2BP) showed no significant correlation with the well-established CKD markers and cytokines.

The raw MRM data and gel images were deposited at jPOST database [25] under the project numbed as JPST000578 and JPST000579.

## 4. Discussion

Modern diagnostics of CKD and monitoring of its course are based on the measurement of serum creatinine concentrations and subsequent calculation of the glomerular filtration rate [3,4]. The disadvantage of this method is its poor effectiveness in diagnosing early CKD. There are a number of studies where potential protein biomarkers of CKD were proposed and evaluated in the serum or plasma of patients [7,8,11,12,13,14]. Peptide biomarkers can also provide insight into CKD diagnosis and progression. In a recent study, 273 specific urinary peptides (CKD273 classifier) were identified as reliable predictors of CKD progression [26] and cardiovascular events in CKD [27]. In this study, we hypothesized that if some proteins showed significant quantitative changes in patient serum and correlated with well-established markers, then they would be potential biomarkers for CKD diagnosis. The first stage of our research involved the screening of serum proteins with differential expressions in patients with CKD and healthy individuals using the 2D-DIGE approach. Twenty-one proteins from the 2D dataset were selected as potential protein candidates for biomarkers of the disease (Table 2). AAT, APOA4 and VIL1 concentrations have previously been shown to increase in blood plasma as CKD progresses [28,29,30,31]. Our data confirm the increased blood serum concentrations of these proteins in patients; whereas only serum AAT values correlated with elevated levels of creatinine and cytokines. Our target proteomics results are compared with previously reported data from studies of different renal pathologies in Table 3.

AAT, which is supposed to have anti-inflammatory properties, belongs to the group of serine proteinases [27]. Exogenous AAT inhibited apoptosis and inflammation in renal reperfusion [28] and reduced the urine kidney injury molecule-1 (KIM-1) concentration [30]. Several studies have demonstrated increased blood plasma AAT concentrations in hypoxia [31] and acute myocardial infarction [48,49].

Based on the MRM results, APOE concentrations were significantly higher in the group of patients with CKD, whereas serum APOA1 levels showed no changes. However, in previously reported studies, plasma APOE and APOA1 levels differed for patients with and without CKD, apparently depending on the disease stage and therapy [35,50,51]. In particular, hemodialysis and pharmacotherapy might have an impact on the levels of these proteins.

High antigen KI-67 and CUL5 expression levels in tissues are associated with cancers [44,52,53,54,55,56]. We observed increased serum concentrations of these proteins in patients with CKD in this study. Furthermore, we saw increased levels of other intracellular proteins such as NKRF, CCDC171, CAPRI, CCDC43, APPBP2, IGSF22 and PKCE, which might be associated with renal tissue necrosis and, thus, with disease progression. The literature on these proteins in CKD is limited and/or contradictory. IGSF22 is known to be similar to cytoskeletal proteins in its structure, with a one-nucleotide substitution in the IGSF22 gene associated with the development of renal carcinoma [41]. The information on PKCE is controversial; protein kinase activation in proximal nephron cells leads to impaired functioning of the mitochondria, oxidative stress, energy deficiency and cell death [57]. At the same time, there is evidence that PKCE has protective functions against ischemic injury in other tissues, particularly in the myocardium and neurons [58,59,60,61]. NKRF is a transcriptional repressor of NF-kappa-B responsive genes [62]. Increased expression of NKRF transcription factor was shown to upregulate IL-1-induced secretion of IL-8 [63]. In our study, increased serum levels of IL-1 and IL-8 were also demonstrated, suggesting that NKRF could synergize with IL-1 to induce IL-8 expression. It should be noted that serum HSP90B2 concentration positively correlated with levels of creatinine, urea and a number of cytokines. However, the data on changes of serum HSP90B2 in various pathologies are presently lacking. We have evaluated key complement system proteins known to be important components of innate immunity and to play important roles in body defense, inflammation, tissue regeneration and other physiological processes. We found that serum C1S, C1QC and C4 concentrations increased two to three times, and that of C1R was elevated four times in patients with CKD, as compared to the control subjects. Increased levels of these proteins might indicate the activation of the complement system under the classic pathway. It is noteworthy that concentrations of lectin pathway activators such as FCN3, Gal-3 and MASP2 did not show any significant difference between the groups, although the MBL2 concentration was twice as high in patients with CKD. MBL2 has been previously shown to be capable of binding with apoptotic and necrotic cells, thereby promoting the activation of phagocytosis of dying cells [64].

Serum concentrations of complement system inhibitors factor H and C4BP were elevated two and four times, respectively, in CKD patients. We found significantly elevated concentrations (two times) of late lytic cascade proteins such as C5, C8A and C8B, and both C3 and C9 were more than four times higher in CKD patients. These data are in consistent with previously reported results about higher plasma MAC (C5b-9) levels in patients with renal diseases [45,46].

It is remarkable that the serum concentration of the subunit С8G did not elevate following the increase of C8A and C8B subunits constituting a single С8 protein. Unlike C8A and C8B, the С8G subunit is a member of the lipocalin family. As Lovelace et al. have previously reported [65], С8g is not involved in the formation of a membrane attack complex, but it only enhances its activity [65]. Perhaps, the subunit С8G has a regulator function as a complement system inhibitor. Furthermore, it has been suggested that decrease in serum С8G might be specific for other chronic diseases.

Increased blood serum levels of cytokines such as IL-4, IL-5, IL-6, IL-10 and IL-13, G-CSF, eotaxin and MIP-1β might indicate the activation of Th2 cells responsible for the development of humoral immune responses [66,67]. It should be noted that increased blood levels of cytokines involved in the activation of Th1 cells as well as IL-2, GM-CSF, TNF-α, IL-12, RANTES, MIP-1α and IL-18 were previously observed in patients with CKD [68,69]. Simultaneous activation of Th1 and Th2 cells was shown in various renal pathologies [69,70,71] and in a number of other diseases [72,73,74]. Moreover, significantly increased IL-9 might suggest ongoing differentiation of Т-cells into a Th9 population [73].

## 5. Conclusions

Early diagnosis of renal dysfunction is essential to improve disease progress and survival of patients with CKD. In this research we quantified differentially expressed proteins and complement components using the MRM approach and measured serum concentrations of cytokines, chemokines and growth factors using the multiplex Luminex xMAP technology in patients with CKD and healthy subjects.

Our results correlate well with the data obtained by other researchers in that blood APOA4, AAT, VIL1, complement component and cytokine concentrations increased in patients with renal disorders [8,11,12,13,14,28,32,33,34,43,69,70,71]. Moreover, we found elevated serum concentrations of potential oncomarkers (CUL5, antigen KI-67) and other intracellular proteins (NKRF, CAPRI, IGSF22, APPBP2, CCD171 and CCD43) in patients with CKD. The reasons for this increase in blood serum and the role they play in renal tissue injury are not clear and require further investigations.

Patient serum levels of AAT, IGS22 and HSP90B2 had a greater than four-fold change. In addition, the data analysis showed a mild correlation between increased serum concentrations of AAT and HSP90B2 in patients with CKD and well-established markers of CKD such as creatinine and urea. Thus, we suggest that proteins AAT and HSP90B2 might be associated with kidney diseases and might be potential markers of CKD. Further investigations of these proteins as early biomarkers are needed to elucidate their clinical usefulness.

## Figures and Tables

**Table 1:** Characteristics of the study population.

Parameters *	CKD Patients
Age (y)	53.0 ± 16.2
Sex (males/females)	18/8
eGFR (mL/min/1.73 m^2^)	14.7 ± 3.1
Diagnosis of CKD (number of patients)	26
Chronic glomerulonephritis	18
Diabetes	3
Chronic gouty nephropathy	2
Others or unknown	3
Body mass index (kg/m^2^)	25.8 ± 5.6
Systolic blood pressure (mmHg)	128.7 ± 23.2
Diastolic blood pressure (mmHg)	79.2 ± 18.3
Creatinine (mg/dL)	7.25 ± 3.8
Urea (mg/dL)	135.0 ± 70.2
Albumin (g/dL)	3.8 ± 0.5
Total cholesterol (mg/dL)	177.8 ± 40.2
HDL cholesterol (mg/dL)	52.1 ± 28.4
LDL cholesterol (mg/dL)	110.6 ± 45.6
Triglyceride (mg/dL)	141.9 ± 50.1

* Abbreviations: CKD—chronic kidney disease; eGFR—estimated glomerular filtration rate; HDL—high-density lipoprotein; LDL—low-density lipoprotein.

**Table 2:** Multiple reaction monitoring (MRM) quantification proteins list.

n/n	Protein	UniProt Accession Number	Target Peptide Sequence	MRM Transition Q1	MRM Transition Q3	Product Ion
1	Immunoglobulin superfamily member 22 (IGSF22)	Q8N9C0	EDSGLILLK	494.3	743.5	y7
2	T-complex protein 1 subunit delta (CCT4)	P50991	LVIEEAER	479.8	655.4	b6
3	Cullin-5 (CUL5)	Q93034	EAFQDDPR	489.2	777.4	y6
4	Apolipoprotein A-IV (APOA4)	P06727	LAPLAEDVR	492.3	589.3	y5
5	Apolipoprotein E (APOE)	P02649	LGPLVEQGR	484.8	588.3	y5
6	Apolipoprotein A-I (APOA1)	P02647	QGLLPVLESFK	615.9	819.5	y7
7	Coiled-coil domain-containing protein 43 (CCDC43)	Q96MW1	LEALGVDR	436.7	446.2	y4
8	Coiled-coil domain-containing protein 171 (CCDC171)	Q6TFL3	TLQEALEK	466.3	830.5	y7
9	Putative endoplasmin-like protein (HSP90B2)	Q58FF3	FDDSEK	370.7	478.2	y4
10	Plasminogen (PLG)	P00747	LSSPAVITDK	515.8	769.4	b8
11	Phospholipase B1 (PLB1)	Q6P1J6	TETLDLR	424.2	445.2	b4
12	LIM and cysteine-rich domains protein 1 (LMCD1)	Q9NZU5	YSTLTAR	406.2	465.2	b4
13	Alpha-1-antitrypsin (AAT)	P01009	LSITGTYDLK	555.8	797.4	y7
14	Villin-1 (VIL1)	P09327	AFEVPAR	395.2	442.3	y4
15	NF-kappa-B-repressing factor (NKRF)	O15226	EIPPADIPK	490.3	736.4	b7
16	Amyloid protein-binding protein 2 (APPBP2)	Q92624	VVVDVLR	400.3	700.4	y6
17	Serine/threonine-protein phosphatase with EF-hands 2 (PPEF2)	O14830	SLPSSPLR	428.7	472.3	y4
18	Ras GTPase-activating protein 4 (CAPRI)	O43374	DELDLQR	444.7	531.3	y4
19	Cytoskeleton-associated protein 2-like (CKAP2L)	Q8IYA6	QFVGETQSR	526.3	776.4	y7
20	Protein kinase C epsilon type (PKCE)	Q02156	QINQEEFK	518.2	613.2	b5
21	Antigen KI-67	P46013	EDSTADDSK	484.1	504.1	b5
22	Complement factor H (CFH)	P08603	NGFYPATR	463.2	607.3	y5
23	Complement C4 (C4A, C4B)	P0C0L4 P0C0L5	LTSLSDR	396.2	577.3	y5
24	Ficolin-3 (FCN3)	O75636	VELEDFNGNR	596.8	722.3	y6
25	C4B-binding protein alpha chain (C4BPA)	P04003	TWYPEVPK	510.3	569.3	y5
26	Complement C1R subcomponent (C1R)	P00736	GGGALLGDR	408.2	460.3	y4
27	Complement C1S subcomponent (C1S)	P09871	LLEVPEGR	456.8	686.3	y6
28	Complement C1q subcomponent subunit C (C1QC)	P02747	FQSVFTVTR	542.8	623.4	y5
29	Complement С3 (C3)	P01024	IWDVVEK	444.7	474.3	y4
30	Complement С5 (C5)	P01031	GTVYNYR	436.7	452.2	y3
31	Complement component C8 alpha chain (C8A)	P07357	STITYR	370.7	552.3	y4
32	Complement component C8 beta chain (C8B)	P07358	EYESYSDFER	662.8	672.3	b5
33	Complement component C8 gamma chain (C8G)	P07360	QLYGDTGVLGR	589.8	678.3	b6
34	Complement С9 (С9)	P02748	VVEESELAR	516.3	833.4	y7
35	Mannose-binding protein C (MBL2)	P11226	NAAENGAIQNLIK	678.4	869.4	b9
36	Mannan-binding lectin serine protease 2 (MASP2)	O00187	WPEPVFGR	494.3	609.3	b5
37	Galectin-3 (Gal-3)	P17931	LDNNWGR	437.7	671.3	y6
38	Galectin-3-binding protein (M2BP)	Q08380	VEIFYR	413.7	727.4	y5

**Table 3:** Lists of elevated serum proteins from MRM data analysis compared to the previously reported results.

n/n	Protein	Fold Change	SC between Protein and Creatinine	SC between Protein and Urea	Reference	Study Population	Results
1	APOA4	3.4 * ↑	0.07	0.19	[32]	345 CKD patients with type 2 diabetes	Increased plasma level of APOA4
					[33]	177 CKD patients	Increased serum level of APOA4 were significant predictors of disease progression
					[34]	6220 participants of general population	Increased serum level of APOA4 were significant predictors of disease progression
2	APOE	2.1 ** ↑	0.30	0.30	[35]	117 CKD patients	APOE was a negative predictor of eGFR reduction rate
					[36]	109 HD patients	APOE were significantly decreased
					[8]	90 CKD patients	Elevated level of APOE in plasma of patients with CKD 1-2 stages
					[37]	301 HD patients	HD patients had a significantly lower prevalence of the E4 allele and greater levels of APOE
					[38]	7 CKD patients	Increased plasma level of APOE
3	APOA1	1.6 * ↑	−0.16	−0.07	[39]	17,315 participants of the general population	Higher serum APOA1 was associated with lower prevalence of CKD
					[40]	50 patients with CKD and 198 patients on HD therapy	CKD was found to be associated with highly significant reductions in plasma APOA1
					[8]	90 CKD patients	No differences between plasma APOA1 level of patients with CKD 1-2 stages and healthy voluntaries
					[11]	76 patients who received initial insertion of PD	APOA1 showed enhanced levels in PD effluents of patients with high transporter
4	IGSF22	4.5 ** ↑	0.34	0.35	[41]	7 patients with clear cell carcinoma	Found in a renal cell carcinoma sample; somatic mutation
5	HSP90B2	4.0 ** ↑	0.55 **	0.56 **	-	-	-
6	AAT	8.7 ** ↑	0.44 *	0.41	[12]	12 non-diabetic ESRD patients	HD patients had altered plasma profiles of AAT isoforms
					[31]	63 patients with primary membranous nephropathy	Increased urinary level of AAT
					[42]	103 HD patients	Higher serum AAT levels select the HD patients with severe inflammation from those without
7	VIL1	2.6 * ↑	0.08	0.18	[43]	3 patients with AKI after liver transplantation	VIL1 is released in plasma during AKI and shows potential as an early marker for proximal tubular injury
					[29]	3 renal transplant recipients	VIL1 concentrations in the urine up to 20 mg/I
8	Antigen KI-67	3.2 * ↑	0.31	0.31	[44]	351 patients with clear cell carcinoma	Ki-67 are significant prognostic factors of clear cell carcinoma
9	CFH	2.7 * ↑	0.43 *	0.43 *	[45]	63 patients with RD	Urinary CFH levels were significantly higher in patients
10	C4A	2.8 ** ↑	0.42	0.45 *	[38]	7 CKD patients	Increased plasma level of CA4
					[13]	90 patients with CKD	Increased plasma level of CA4
11	C4BPA	4.5 ** ↑	0.3	0.38	-	-	-
12	C1R	4.1 ** ↑	0.48 *	0.49 *	[14]	29 patients with CKD	Increased plasma level of C1R
13	C1S	2.1 ** ↑	0.51 *	0.51 *	[14]	29 patients with CKD	Increased plasma level of C1S
14	C1QC	3.7 ** ↑	0.46 *	0.50 *	[46]	62 diabetic patients	No difference
15	C3	4.7 ** ↑	0.48 *	0.50 *	[11]	76 patients who received initial insertion of PD	C3 showed enhanced expression in PD effluents of patients with high transporter
					[38]	7 CKD patients	Increased plasma level of C3
16	C5	2.2 * ↑	0.11	0.15	[45]	63 patients with RD	Increased urinary MAC (SC5b-9)
17	C8A	2.4 ** ↑	0.48 **	0.47 *	[45]	63 patients with RD	Increased urinary MAC (SC5b-9)
18	C8B	2.7 ** ↑	0.25	0.38	[45]	63 patients with RD	Increased urinary MAC (SC5b-9)
19	C8G	3.1 ** ↓	−0.41	−0.63 *	[38]	7 CKD patients	Decreased plasma level of C8G
20	С9	11 ** ↑	0.58 **	0.62 **	[45]	63 patients with RD	Increased urinary MAC (SC5b-9)
					[47]	53 patients with different nephropathy	Urinary C9 was elevated in MCD, MN and FSGS groups compared with in IgA nephropathy and healthy controls
21	MBL2	3.4 ** ↑	0.18	0.18	[46]	62 diabetic patients	MBL was found to increase with the progression of DN
22	CUL5	3.3 ** ↑	0.23	0.29	-	-	-
23	PKCE	3.2 ** ↑	0.27	0.28	-	-	-
24	CCDC43	2.2 * ↑	0.18	0.22	-	-	-
25	CDC171	3.1 ** ↑	0.33	0.38	-	-	-
26	CAPRI	2.1 * ↑	0.18	0.23	-	-	-

* *p* < 0.05, ** *p* < 0.005, ↑—increased in CKD patients, ↓—decreased in CKD patients. Abbreviations: SC—Spearman’s rank correlation coefficient, HD—hemodialysis, PD—peritoneal dialysis, ESRD—end-stage renal disease, AKI—acute kidney injury, RD—renal disease, MCD—minimal change disease, MN—membranous nephropathy, FSGS—focal segmental glomerulosclerosis, DN—diabetic nephropathy.

## Supplementary Materials

The following are available online at https://www.mdpi.com/2218-273X/10/2/257/s1, Table S1: List of peptides for MRM analysis, Table S2: Proteins differentially expressed between CKD and control groups, Table S3: Average mean, standard error and standard deviation, Table S4: Significant differences, P-value <0.05, Table S5: Spearman’s rank correlation.
